# Optical Fourier Surfaces for Integrated Photonics

**DOI:** 10.1021/acsnano.6c04440

**Published:** 2026-06-09

**Authors:** Daniel Petter, Fabian Kaufmann, Daniel Chelladurai, Manuel Kohli, Andreas Maeder, Yannik M. Glauser, Nolan Lassaline, J. J. Erik Maris, Juerg Leuthold, Rachel Grange, David J. Norris

**Affiliations:** † Department of Mechanical and Process Engineering, 27219 ETH Zurich, 8092 Zurich, Switzerland; ‡ Department of Physics, ETH Zurich, 8093 Zurich, Switzerland; § Department of Information Technology and Electrical Engineering, ETH Zurich, 8092 Zurich, Switzerland

**Keywords:** thermal scanning-probe lithography, integrated photonics, Bragg filter, high-Q cavity, thin-film lithium niobate, second harmonic generation

## Abstract

Integrated photonics has enabled the miniaturization and development of classical as well as quantum-optical technologies. However, traditional lithographic techniques limit the required optical elements to binary height profiles. By relaxing these constraints, grayscale fabrication methods have the potential to deliver more efficient and compact devices. In contrast to binary profiles, wavy surfaces (also known as optical Fourier surfaces, OFSs) only introduce spatial frequencies that are required for their functionality, yielding more control over the optical response. In this work, we demonstrate photonic integrated circuits with grayscale OFS elements in state-of-the-art material platforms, including silicon-on-insulator and thin-film lithium niobate. Using thermal scanning-probe lithography and dry etching, the OFSs are written and transferred with high fidelity. We employ an intuitive and straightforward design scheme to create a series of devices that exploit the capabilities of wavy height profiles. First, we fabricate sinusoidal single- and multiband Bragg reflectors in silicon waveguides with an extinction ratio of up to 44 dB at 1550 nm. Second, cavities in lithium-niobate waveguides are fabricated with a high quality factor of 1.6 × 10^5^ and a theoretical modal volume of 2.4­(λ/*n*)^3^. Finally, we exploit this high-quality-factor cavity and the large optical nonlinearity of lithium niobate to produce frequency-doubled light via second harmonic generation.

## Graphical abstract

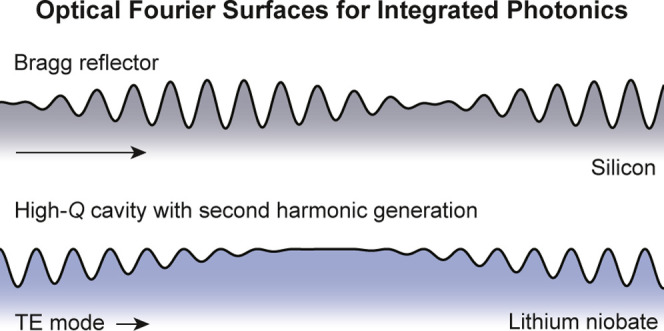

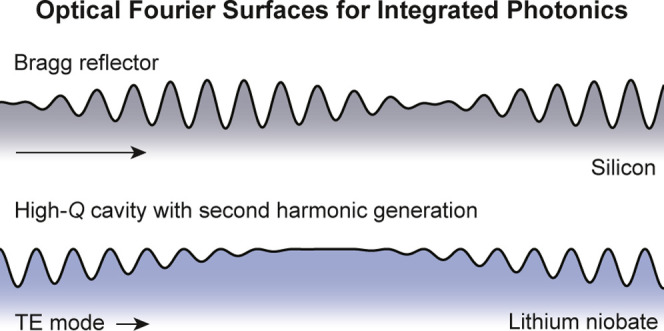

The miniaturization of optical structures has allowed the field of integrated photonics to mature and innovate.^1–3^ It has led to many pioneering technologies, including quantum communication,^4,5^ on-chip medical diagnostics,^6^ and (quantum) optical computing.^7,8^ To advance these and other areas, simultaneous improvement in material properties, designs, and nanofabrication is required. In particular, photonic structures (cavities, gratings, waveguides, etc.) must be fabricated on-chip either via electron-beam or ultraviolet lithography. While these processes offer a viable and scalable approach, they typically produce devices with binary surface profiles that contain only two depth levels. Such binary fabrication constraints can limit the development of photonic integrated components and hinder their optimization.

Specifically, modes traveling through waveguides with binary profiles experience discontinuities in the effective permittivity. These can lead to undesired mode couplings that decrease the performance of photonic integrated devices.^9^ For example, they may cause significant back reflections in broadband grating couplers,^10,11^ complicate controlled mode conversions,^9^ and introduce sidelobes in the response of spectral filters.^12^ Another limitation of binary surfaces is that gradual tapering of the height profile is not possible. This restricts the height of the photonic device to the intrinsic layer thickness of the guiding material on the chip. Vertical tapering would allow different photonic-layer thicknesses to be used and reduce optical losses when transitioning between them. While binary lithographic techniques can avoid the above challenges by incorporating subwavelength gratings,^3^ such structures require high resolution and accurate positioning, making them prone to fabrication errors.^13^ Thus, a need exists for alternative fabrication methods that can produce smooth, wavy photonic elements.

One approach is to exploit grayscale lithography—a class of fabrication methods that produces surfaces with many depth levels. These include grayscale electron-beam lithography (gEBL), two-photon grayscale lithography (2PGL), and laser interference lithography (LIL). Each of these approaches creates a pattern in a resist layer that is then transferred into the photonic material below. While various grayscale structures can be produced, these lithographic techniques have not yet reached the control required (in terms of depth, resolution, and smoothness) to enable high-performance integrated photonic devices. In particular, proximity effects in gEBL^14,15^ and optical diffraction in 2PGL and LIL^16,17^ limit the structures that can be fabricated. This motivates the exploration of alternative fabrication methods that do not suffer from these limitations.

We have recently shown that thermal scanning-probe lithography (tSPL) can provide sufficient control to produce smooth, wavy photonic elements (also known as optical Fourier surfaces, OFSs) with nanometer precision.^18,19^ A grayscale lithographic mask is created by scanning a hot, sharp silicon tip across a thermally sensitive resist, locally removing material to a desired depth (Figure 1a). Structures in the resist can be patterned in a field size up to 50 × 50 μm^2^ with a depth up to ∼300 nm, a lateral resolution better than 50 nm, and a maximum slope of ∼1.^20,21^ This mask is then transferred to the underlying photonic layer via dry etching (Figure 1b). Depending on the etching recipe, the depth of the initial pattern can be amplified.^22^ However, while this general route has been used to produce simple surface structures in several materials relevant for photonic technologies (silicon,^23–25^ silicon nitride,^26^ silicon dioxide,^22^ and hexagonal boron nitride^20^), the full capabilities of tSPL have not yet been exploited to create functional elements that are embedded in photonic integrated circuits.

**Figure 1:**
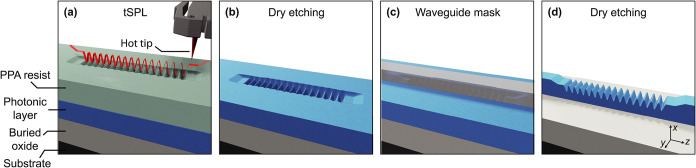
Fabrication scheme for wavy integrated photonic circuits. (a) A thin layer of PPA resist is spin coated onto a wafer, consisting of a photonic layer, a buried oxide layer, and a substrate. Using tSPL, a pattern is written with a hot tip into the PPA layer to create a grayscale lithographic mask. (b) The grayscale pattern is dry etched from the PPA into the underlying photonic layer. (c) A binary lithographic mask is written via electron-beam lithography, covering the grayscale pattern and defining the integrated photonic circuit. (d) The OFS, a photonic waveguide with a grayscale profile, is obtained with a second dry-etching process.

Here, we utilize tSPL to incorporate OFSs—including optical filters, grating couplers, and resonators—in photonic integrated circuits. We demonstrate structures that span the full thickness of the photonic layer for three important material platforms: silicon (Si), lithium niobate (LiNbO_3_), and silicon nitride (SiN_*x*_, see Section S1 in the Supporting Information). We design and fabricate grayscale spectral filters and high quality-factor (*Q*) cavities using silicon-on-insulator (SOI) and lithium-niobate-on-insulator (LNOI) substrates, respectively. For the cavities, we leverage their high *Q* and the large optical nonlinearities of LiNbO_3_ to generate second harmonic light on chip. By demonstrating such functional OFSs, we show that tSPL can provide high-performance integrated photonic circuits that can avoid the limitations of binary profiles.

## Results and Discussion

### Device Fabrication

The fabrication scheme to obtain wavy integrated photonic structures is depicted in Figure 1. We fabricate our devices starting with commercially available wafers, which consist of a photonic layer (e.g., Si), a buried oxide layer (SiO_2_), and a substrate (Si). A thermally sensitive polymer resist, poly­(phthalaldehyde) (PPA), is spin coated on this stack. By patterning the PPA layer with tSPL, a grayscale lithographic mask is created (Figure 1a).^18^ This grayscale mask is then dry etched with an inductively coupled plasma (ICP) reactive-ion etching (RIE) tool into the underlying photonic layer. In this work, we chose the etching recipe such that the depth profile in the PPA mask is transferred into the photonic layer with negligible amplification (Figure 1b). After a cleaning step, the grayscale height profile in the photonic layer is revealed. To define the waveguides in the photonic integrated circuit, a binary lithographic mask is then written with electron-beam lithography (Figure 1c). A second dry-etching process followed by a cleaning step creates the final grayscale structure (Figure 1d).^27–30^ Further details about the fabrication procedures are provided in the Methods and Section S1 in the Supporting Information.

The above scheme works for any material platform, given that suitable dry-etching conditions exist to transfer the pattern from PPA into the targeted photonic layer. For Si and SiN_*x*_, it was possible after extensive parameter studies to develop reliable etching protocols. Notably, these allowed the patterns to be transferred into the photonic layer without any significant deformation. For LiNbO_3_, which is known to be challenging to etch, direct transfer from PPA into LiNbO_3_ was not possible. Etching recipes for LiNbO_3_ typically exploit argon ions^31,32^ or fluorine-based gases^33^ at high kinetic energies. However, the soft PPA mask is etched uncontrollably under these conditions. To overcome this problem, we modified the fabrication procedure in Figure 1 by adding an amorphous SiN_*x*_ layer between the LiNbO_3_ and PPA. In other words, our process uses a PPA/SiN_*x*_/LiNbO_3_ stack. The tSPL pattern in the PPA is first transferred into the SiN_*x*_ layer, which serves as an intermediate hard mask. The final grayscale pattern is then obtained by ion milling the SiN_*x*_ profile into the LiNbO_3_ (Figure S1 in the Supporting Information). This modified procedure can be applied to other difficult-to-etch material platforms.

Using these etching recipes, our fabrication scheme allows multiple OFSs with different functionalities to be combined into one photonic integrated circuit. Here, we demonstrate circuits that consist of three grayscale devices connected by waveguides. Specifically, each circuit comprises an OFS device, such as an optical filter or a high-*Q* cavity, connected to two OFS grating couplers for bringing light into and out of the waveguide.^25^ The grating couplers are described in Section S2 in the Supporting Information.

### Bragg Reflectors in SOI

In integrated photonics, the Si material platform is by far the most advanced because of its compatibility with manufacturing processes perfected for the mass production of electronic chips.^34^ Si is also highly transmissive at the wavelengths commonly used for telecommunications (1310 and 1550 nm). Consequently, Si photonic integrated circuits have been developed to manipulate optical signals.

One common operation in such devices is to filter out a certain wavelength range from the transmitted light, for example, by incorporating a Bragg reflector (BR). Fourier optics presents an elegant approach to design grayscale BRs with one or more stop bands.^35,36^ For example, to create a BR with a single stop band, one can use a sinusoidal height modulation of the form

1 $$h\left(z\right)=h_{0}+A\cdot \cos\left(\frac{2\pi }{\Lambda }z+\varphi \right)$$

following the coordinate system in Figure 1d, where *h*_0_ is the mean height, Λ the period, *A* the amplitude, and φ the phase. To a good approximation, this modulation in height leads to a sinusoidal modulation in effective permittivity (see Section S3 in the Supporting Information)

2 $$\epsilon_{\mathrm{eff}}\left(z\right)=\epsilon_{\mathrm{eff},0}+\Delta \epsilon \cdot \cos\left(\frac{2\pi }{\Lambda }z+\varphi \right)$$

where ϵ_eff,0_ is the effective waveguide permittivity at height *h*_0_ and Δϵ the amplitude of the modulation. Thus, light traveling through the waveguide experiences a periodically modulated permittivity, which introduces a single stop band. Specifically, the resulting BR reflects light around a free-space wavelength, $\lambda_{0}=\sqrt{\varepsilon_{\mathrm{eff},0}}\Lambda$ .

To demonstrate the feasibility of this approach, we fabricated a BR for λ_0_ = 1550 nm incorporated into an SOI waveguide. The scanning electron microscopy (SEM) image in Figure 2a shows the beginning and end of the fabricated BR. The depth profile of the waveguide, recorded with atomic force microscopy (AFM), is given in Figure 2b. Importantly, our fabrication scheme (Figure 1) allows additional design features to be implemented without extra processing steps. First, we added a vertical taper, which reduces the waveguide height to a regime where eq 2 is a good approximation. In addition, it reduces losses, as explained in the next paragraph. Second, we gradually decreased the amplitude of the sinusoidal modulation toward the beginning and end of the BR (Figure 2b). Such an apodization minimizes unwanted back reflections at its entrance and exit, because it provides an adiabatic transition between the effective permittivities of the waveguide and BR.

**Figure 2:**
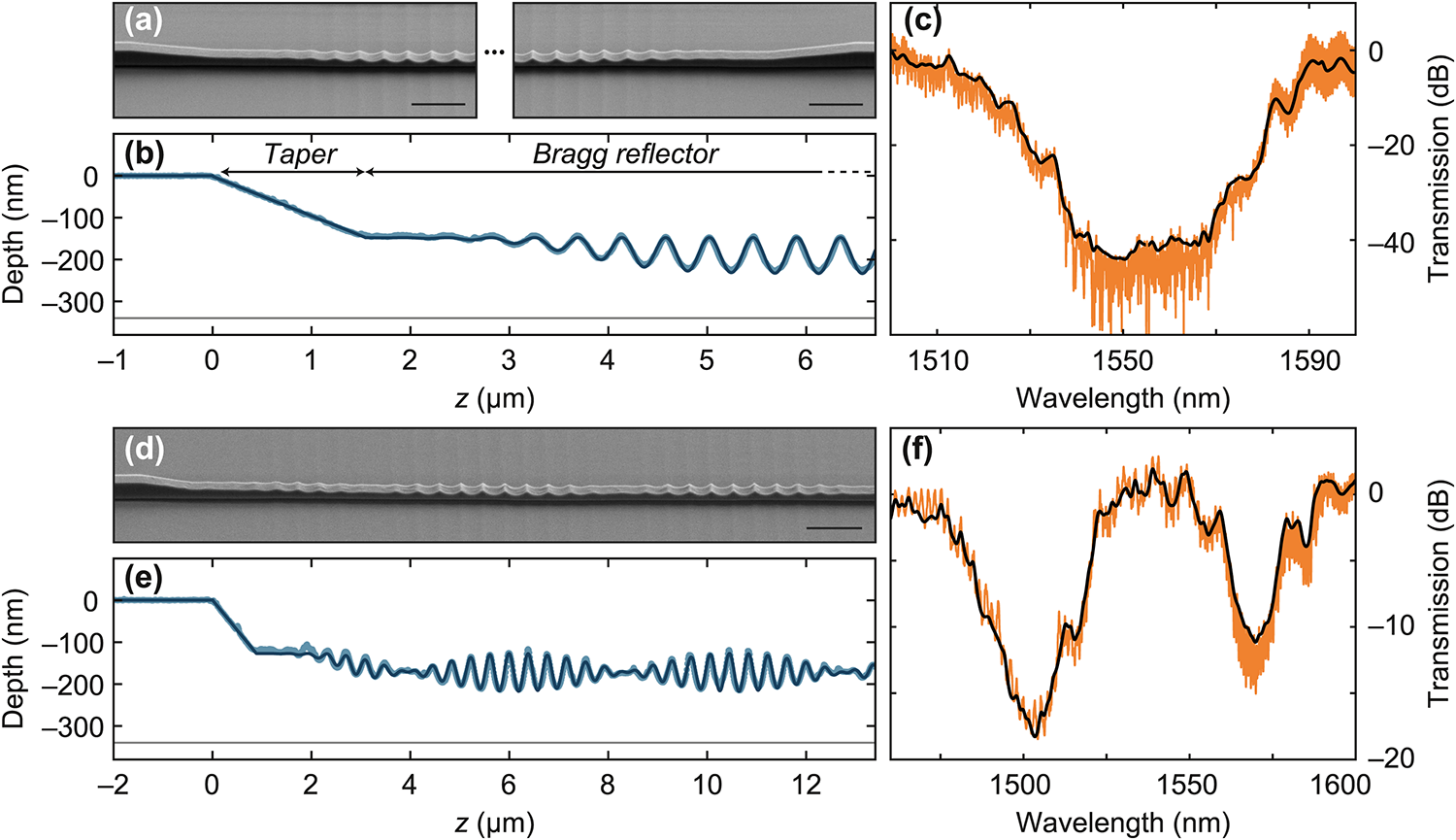
Bragg reflectors for silicon photonics. (a) Scanning electron micrograph of a single-sinusoidal BR on an SOI waveguide with a transversal width of 450 nm. The reflector is 44.2 μm long and contains 100 sinusoidal periods. (b) Atomic force microscopy scan (blue dots) of the height profile of the BR in panel (a). The profile contains a vertical taper followed by the BR. Within the BR, the amplitude of the modulation gradually increases to a maximum of 40 nm. The black line is the desired functional form of the pattern that is fit to the measured profile (see Section S1 in the Supporting Information). The root-mean-square (RMS) error between the fitted function and the measured profile is 4.4 nm (excluding unpatterned regions at zero depth). (c) Experimental transmission spectrum of the device in panel (a). The BR introduces a stop band around 1550 nm with a maximum extinction ratio of 44 dB. The transmission is normalized to a reference device without a BR. The orange line indicates the normalized measured data, while the black solid line is a spline fit of the data (see Section S1 in the Supporting Information). (d)–(f) Same as in panels (a)–(c) but for a BR with a height profile containing two superimposed sinusoids, introducing two stop bands. All optical measurements were done with a poly­(methyl methacrylate) (PMMA) cladding to protect the devices. All scalebars are 1 μm.

The transmission spectrum of the OFS BR is shown in Figure 2c. At λ_0_ = 1550 nm, the device has an extinction ratio of 44 dB. While this is almost an order of magnitude better than previously reported values for BRs with sidewall corrugations (i.e., sinusoidally modulated widths),^37^ a direct comparison is challenging due to differences in the BR lengths, modulation amplitudes, and photonic-layer thicknesses. Our extinction ratio is also improved by our ability to taper the thickness to 150 nm. In this regime, no transverse-magnetic (TM) modes are supported, and losses caused by coupling to these modes due to polarization scattering are avoided. For BRs with sidewall corrugations, shallow holes etched in the center of the waveguide similarly minimize coupling to TM modes, improving the extinction ratio.^38^ However, the addition of these holes also increases propagation losses and requires an extra etching step in the fabrication process.

Multiple stop bands can be introduced by adding more than one sinusoid (Fourier component) to eqs 1 and 2. This leads to several superimposed oscillations in the permittivity. We created a BR with a height profile containing two sinusoidal waves (Figure 2d,e) that introduces two stop bands in the transmission spectrum (Figure 2f). The OFS BR suppresses the transmission at 1500 and 1570 nm by 17 and 12 dB, respectively. To stay in the regime in which eq 2 is a good approximation, we kept the height profile in the same range as the single-stop-band BR. Consequently, each sine wave has a smaller amplitude. This reduces their individual contributions, leading to smaller extinction ratios than that for a BR with a single stop band. Currently, our extinction ratios are limited by the maximum pattern length that can be written with tSPL, but they can be improved by increasing the device size by stitching multiple patterns in series.^19,39^ All together, these results demonstrate the feasibility to fabricate wavy integrated photonic devices with tailored spectral transmission.^12^

### High-*Q* Cavities in LNOI

LiNbO_3_ has long been exploited in electro-optic modulators.^40^ However, its use in photonic integrated circuits has been limited until recently.^29,31,41–43^ This is despite its advantageous optical properties, including a broad transmission window (350 nm–5 μm), a high nonlinear coefficient [χ^(2)^ = 30 pm V^–1^], and a large piezoelectric response (*C*_33_ = 250 C m^–2^).^31,43^ The material platform was long believed to be difficult to pattern, but breakthroughs have led to LiNbO_3_ waveguides with vertical sidewalls sufficiently smooth to obtain high performance.^43^

To show that OFS devices can also be created in LiNbO_3_ using our fabrication approach, we demonstrated an optical cavity in LNOI. We began by designing a topologically protected cavity consisting of two phase-shifted sinusoidal BRs. To identify the topology of these BRs, we inspect the band diagram of a sinusoidal LNOI waveguide and the symmetries of the modes within the bands (Figure 3a). The symmetries at the edges of the Brillouin zone (i.e., at *k*_*z*_ = 0 and 0.5) are affected by the phase φ of the BR (eq 2) with respect to its unit cell (Figure 3b). For a sinusoidal permittivity profile with φ = 0, the symmetry of the lowest band does not change across the Brillouin zone, and hence, its topology is trivial. However, for φ = π, the symmetry of this band inverts across the Brillouin zone, rendering it topological. The bulk–boundary correspondence principle states that at the interface of two photonic crystals with a different topological invariant (Zak phase), a topologically protected state exists within the bandgap.^44,45^ This midgap state is robust to fabrication imperfections, and consequently, has been exploited to build lasers with binary structured waveguides,^46^ micropillar arrays,^47^ and microring resonators.^48^

**Figure 3:**
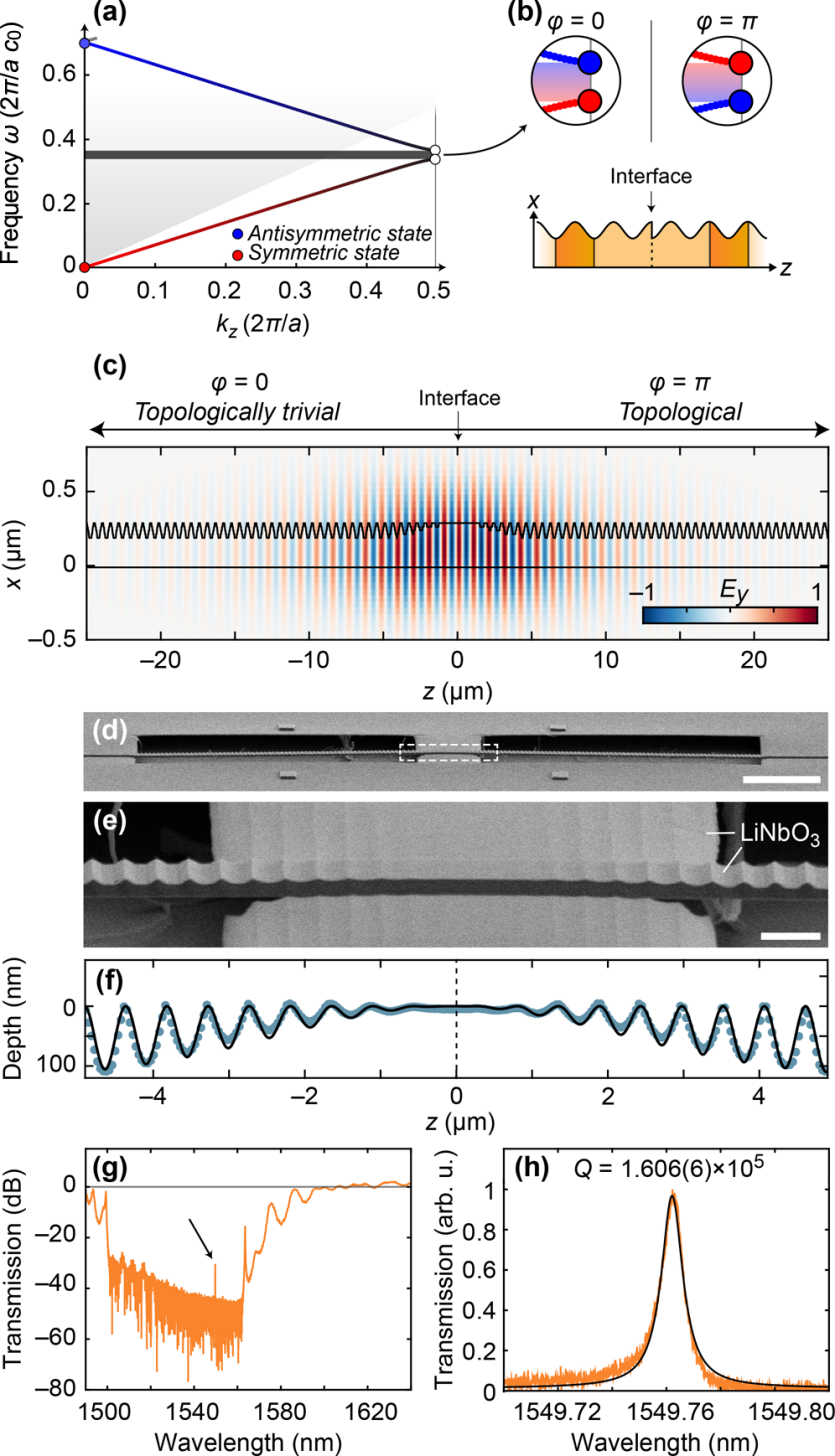
High-*Q* cavities in LNOI. (a) Band structure of a sinusoidally periodic LiNbO_3_ waveguide obtained from a 1D numerical model (see Section S4 in the Supporting Information). The first and second band (colored lines), first bandgap (dark shading), and light cone (light shading) are shown. Colored circles indicate the symmetry of the corresponding band states at the edges of the Brillouin zone (i.e., at *k*_*z*_ = 0 and 0.5). (b) The symmetry of these states depends on the phase φ of the sinusoidal structure (eq 2). When BRs with φ = 0 and π are combined, a topologically protected midgap state exists within the band gap. This state is confined at the interface between the BRs. (c) A central cut of the electric-field distribution of the cavity mode along *y* (*E*_*y*_) computed with a 3D-FDTD simulation at a wavelength of 1538.3 nm. (d) Scanning electron micrograph of the suspended LiNbO_3_ cavity. The structure contains 80 Bragg pairs with a period of 544(1) nm. Scalebar is 10 μm. (e) Central region of the cavity [white dashed box in panel (d)] with a 1 μm scalebar. (f) Atomic force micrograph (blue dots) of the cavity consisting of two phase-shifted BRs. The black line is the desired functional form of the pattern fit to the measured profile (see Section S1 in the Supporting Information). This function was used for the simulation in panel (c). The RMS error between the fit and the measured profile is 8.7 nm. (g) Experimental transmission spectrum of the device. The topologically protected high-*Q* cavity mode inside the bandgap is indicated by an arrow. (h) High-resolution spectrum of the cavity resonance. A *Q* of 1.606(6) × 10^5^ was computed from a Lorentzian fit (solid line).

Using these principles, we designed a topologically protected cavity in *x*-cut LNOI. A 3D finite-difference time-domain (FDTD) simulation of the cavity, which consists of two phase-shifted BRs, is shown in Figure 3c. The computed electric field is overlaid with the height profile. The simulation reveals the cavity mode at the interface of the two BRs (*z* = 0). By fine-tuning the spatial extension of the mode via apodization of the sinusoidal amplitude, scattering losses are minimized. We used this degree of freedom to maximize the *Q* of the cavity.^49^ The calculated mode volume of *V*_m_ = 2.4­(λ/*n*_LiNbO3_),^3^ with λ the wavelength of the mode and *n*_LiNbO3_ the refractive index of LiNbO_3_ in the direction of the electric field, is comparable to values reported for binary LNOI cavities.^50^

SEM and AFM scans of the cavity are presented in Figure 3d–3f, respectively. They show the apodization of the LiNbO_3_ surface at the cavity center and the π phase shift between the BRs. Sinusoids with a period of 544 nm and depth of 110 nm were transferred with high fidelity into the LiNbO_3_ photonic layer. For operation in the visible regime, cavities with a period of 250 nm and depth modulation of ∼50 nm are feasible, assuming the same aspect ratio of the sinusoidal features.

As shown above for SOI, tSPL allows additional design features to be implemented. For LNOI, we use it to create suspended devices. Here, a set of deep, transversal trenches are included in the cavity pattern with tSPL. After dry etching into the LiNbO_3_, these trenches expose the buried SiO_2_ layer around the BRs, leaving a transversal LN bridge at the center of the cavity. A wet etch in buffered hydrofluoric acid then removes the SiO_2_ underneath the LN layer in the cavity region, suspending the cavity, whose center is supported by the LN bridge. See Section S1 in the Supporting Information for further details. Using this approach, the refractive-index contrast between the photonic layer and its surroundings is maximized, leading to an improved *Q*.

Figure 3g shows the transmission of the device, revealing a broad bandgap (with a width of 63.3 nm at −20 dB) opened by the cavity mirrors. Inside the bandgap, a narrow cavity resonance exists at 1550 nm, which is the topologically protected cavity mode. A high-resolution spectrum of the cavity resonance is shown in Figure 3h. From its line width, we find a *Q* of 1.606(6) × 10^5^. This value is comparable to most of the recently published LNOI photonic-crystal cavities.^51–54^ While *Q*s as high as 10^6^ have been reported,^52^ the applied design method makes these cavities sensitive to variations in the fabrication process. Our topologically protected cavity mode is robust to such imperfections, even for high *Q*.

Moreover, our design has not yet been fully optimized. Due to weak coupling from the waveguide to the cavity mode, our device has low transmission at the cavity resonance with a maximum around −30 dB. By tuning the overlap (coupling) between the waveguide mode and the cavity mode via the number of Bragg pairs and the apodization of the height profile, the transmission of the cavity can be maximized while preserving a high *Q* (refs 55,56 ). Furthermore, such apodization can be tuned to maximize the *Q*/*V*_m_ ratio of the cavity,^49,57^ which enhances nonlinear optical conversions of the cavity mode.^52,58,53^

To investigate nonlinear optical effects in the cavity, the emission of frequency-doubled light via second harmonic generation (SHG) was measured. The high *Q* of the resonator enabled the cavity mode to undergo significant SHG. This generated light is diffracted from the cavity in the vertical direction by the BRs, which act as grating couplers for this wavelength. The diffracted light is collected with an optical fiber above the cavity center and recorded with a spectrometer. For these measurements, a LNOI cavity similar to Figure 3 but with *N* = 40 Bragg pairs was fabricated. Because the BR only has half the number of Bragg pairs, the cavity is more transmissive in the bandgap (Figure 4a), yielding a higher coupling of the pump light into the cavity. The spectrum of the detected light is shown in Figure 4b and matches the expected SHG wavelength of the cavity mode (see Section S5 in the Supporting Information). As expected, a quadratic dependence of the SHG signal on the pump power is found (Figure 4c), which confirms the nonlinear effect.

**Figure 4:**
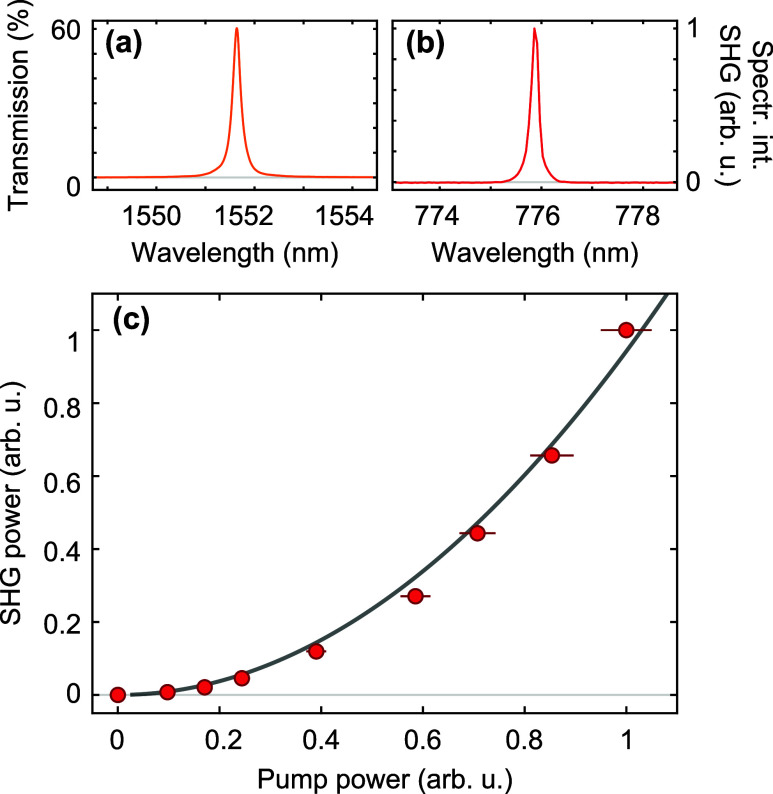
Second harmonic generation in a LNOI cavity. (a) Experimentally measured transmission spectrum of a suspended LiNbO_3_ cavity with 40 Bragg pairs. The cavity supports a mode at λ_res_ = 1551.64 nm with a *Q* of 7940(2). This value is lower than for the cavity shown in Figure 3, but its transmission is higher. (b) Measured SHG spectrum emitted from the cavity, collected with an optical fiber above the cavity center, showing a resonance at λ_SHG_ = 775.87 nm. (c) The power dependence of the SHG light emitted from the cavity mode (red circles) shows a quadratic relationship (solid line) with the input power.

## Conclusion

Leveraging the strengths of tSPL, we demonstrated the feasibility and applicability of OFSs for integrated photonics. Optical elements with a smooth, grayscale height modulation were fabricated in two important material platforms, SOI and LNOI, while showing the fabrication feasibility for SiN_*x*_. The optical structures were more than 100 nm deep and could be transferred with high vertical and lateral resolution. In SOI, we demonstrated single- and multiband rejection filters with an extinction ratio up to 44 dB, which is currently limited by the length of the device. Our design also suppresses polarization scattering, leading to the high extinction ratio reported here. Furthermore, we realized a design scheme based on two phase-shifted sinusoidal BRs to create a topologically protected midgap state in LNOI. The cavity mode had a high *Q* of 1.6 × 10^5^ with a theoretical mode volume of *V*_m_ = 2.4­(λ/*n*_LiNbO3_)^3^. These properties lead to a strong light–matter interaction, which was exploited to perform SHG in the cavity.

The demonstrated optical nonlinearities provide a foundation for novel cavity designs in nonlinear materials. For example, via the nonlinear process of spontaneous parametric down conversion (SPDC), photon pairs could be generated by the cavity mode. With the presented design, these photons could directly leave the cavity, entering the waveguide in the forward direction, while the pump and SHG light would be either reflected or diffracted to free space.^50,59^ Moreover, the profile of the pump field inside the cavity could be shaped to enable phase matching, providing further control over the wave function of the photon pairs. This functionality would be particularly relevant for optical (quantum) computing, where SPDC could be used as a heralded photon source. It could also be employed to incorporate an all-optical nonlinear activation function in integrated photonic neural networks.^60^

We expect that the additional degrees of freedom in OFSs will lead to improved functionality for a broad class of integrated photonic devices. While here we focused on Si, LiNbO_3_, and SiN_*x*_, our developed fabrication scheme with an intermediate hard mask allows for the fabrication of grayscale patterns in other material platforms that are considered hard to etch, such as diamond^61–63^ and barium titanate.^64^ Moving forward, further research efforts should focus on developing design rules to determine when grayscale devices outperform binary ones. For example, echelle grating spectrometers,^65^ optical-computing devices,^66^ and optimized waveguide couplers for single-photon emitters^63,67–69^ are likely to benefit from grayscale surface reliefs. Finally, computational methods relying on inverse design are a promising avenue to determine nonintuitive height profiles that have complex functionalities.

## Methods

### Fabrication of Bragg Reflectors in SOI

A detailed description of the methods is given in Section S1 in the Supporting Information. In short, alignment markers for electron-beam lithography (EBL) and tSPL were first etched into commercially available SOI chips (340 nm Si, 2 μm SiO_2_ on a Si substrate; Soitec). A 260 nm-thick PPA layer (Allresist) was then spin coated, baked for 2 min at 110 °C, and patterned with tSPL to obtain a grayscale mask on top of the Si layer. A dry-etching process, based on a C_4_F_8_/SF_6_/argon gas mixture in an ICP-RIE etching tool (Oxford Plasmalab System 100), was developed to etch the grayscale PPA mask into the Si layer of the chip. The recipe had a typical etch rate in Si of 2 nm·s^–1^ and an etch selectivity of PPA:Si of about 1:1.1. After ultrasonic and plasma cleaning, a 180 nm-thick hydrogen silsesquioxane (HSQ 006, Dow Corning) mask was spin coated, and the waveguide mask was exposed with EBL (EBPG 5200+, Visitec). A subsequent dry-etching process in an ICP-RIE tool was used to etch the photonic integrated circuit into the Si. After removing the residual HSQ mask, the final devices were obtained.

### Fabrication of High-*Q* Cavities in LNOI

Platinum alignment markers for EBL and tSPL were first added to a commercial LNOI chip (300 nm *x*-cut LiNbO_3_, 2 μm SiO_2_ on a Si substrate; NanoLN). A 160 nm-thick SiN_*x*_ layer was then deposited on the chip with plasma-enhanced chemical-vapor deposition (PECVD; Oxford PlasmaPro 100). Subsequently, a 280 nm-thick PPA layer was spin coated, baked at 110 °C for 2 min, and patterned with tSPL. A first dry-etching process, using a C_4_F_8_/SF_6_/argon gas mixture in an ICP-RIE tool, was used to transfer the grayscale PPA mask into the SiN_*x*_ layer. Typical etch rates in PPA of 1.1 nm·s^–1^ and an etch selectivity of PPA:SiN_*x*_ of about 1:1.4 were obtained. After ultrasonic and plasma cleaning, an ion-milling process (Oxford Ionfab 300) with argon ions was used to dry-etch the SiN_*x*_ hard mask into the LiNbO_3_ layer. Then, the chip was cleaned for 5 min in a piranha solution, and the remaining SiN_*x*_ was removed with a 2 min buffered-hydrofluoric-acid (BHF 7:1) etch. To fabricate the waveguide mask, a 500 nm-thick HSQ mask (FOX16, Dow Corning) was spin coated on the chip, exposed with EBL, and etched with argon ions in the ICP-RIE tool to a depth of about 200 nm. To remove redeposited LiNbO_3_, the chip was cleaned for 20 min at 70 °C in KOH (44%). Finally, the chip was immersed for 45 min in BHF, which resulted in the suspended waveguide structures.

### Optical Characterization of the OFS Devices

Both SOI and LNOI devices were characterized with a tunable telecom laser (Keysight N7776C). The light was delivered via optical fibers (SMF28), coupled in and out of the photonic integrated circuits via OFS grating couplers, and detected with a power meter (Keysight N7744C). Each circuit was compared to a reference, which consisted of the grating couplers connected by an unpatterned waveguide. This allowed us to characterize the optical element of interest in the circuit. For optical measurements, the SOI chips were coated with PMMA to protect the photonic circuit outside of the cleanroom. The LNOI chip was not coated with PMMA to avoid damaging the suspended cavities.

### Measurement of SHG Light from LNOI Cavities

SHG light emitted from the LNOI cavities was detected with an optical fiber positioned about 300 μm above the cavity center. A 1555 nm fs-pulsed laser (Menhir Photonics) with a bandwidth of 18.5 nm was used to pump the cavity mode via the waveguides. The collected light above the cavity was sent to a spectrometer (Andor Shamrock 303i). The peak amplitude of the resonance was recorded for different laser pump powers.

## Supplementary Material



## Data Availability

The data that support the findings of this study are available from the corresponding author, D.J.N., upon request.

## Notes

Extended methods; details of OFS grating couplers; effective permittivity in SOI; model of band structure in LNOI; and optical properties of LNOI cavities (PDF)
