# Supplementary material for: Optical Fourier Surfaces for Integrated Photonics
Source: ACS Nano. 2026 Jun 9;20(24):17597–604. doi: 10.1021/acsnano.6c04440 (PMC13296599; doi:10.1021/acsnano.6c04440)
Supplement: Supplementary file 1 [file nn6c04440_si_001.pdf]

Supporting Information for

Optical Fourier Surfaces for Integrated Photonics

Daniel Petter[1], Fabian Kaufmann[2], Daniel Chelladurai[3], Manuel Kohli[3], Andreas Maeder[2], Yannik M. Glauser[1], Nolan Lassaline[1], J. J. Erik Maris[1], Juerg Leuthold[3], Rachel Grange[2], and David J. Norris[1,*]

[1]Optical Materials Engineering Lab, Department of Mechanical and Process Engineering, ETH Zurich, 8092 Zurich, Switzerland

[2]Optical Nanomaterial Group, Institute for Quantum Electronics, Department of Physics, ETH Zurich, 8093 Zurich, Switzerland

[3]Institute of Electromagnetic Fields, Department of Information Technology and Electrical Engineering, ETH Zurich, 8092 Zurich, Switzerland

[*]Email: dnorris@ethz.ch

Contents

S1. Extended Methods 2

S2. OFS Grating Couplers 7

S3. Relationship of the Effective Permittivity and Waveguide Height in SOI 9

S4. Model for the Band Structure of the LNOI Cavity 9

S5. Optical Properties of the LNOI Cavities 10

S6. Supplementary References 12

S1. Extended Methods

Fabrication of silicon-on-insulator Bragg reflectors (BRs). All devices of the silicon-on-insulator (SOI) material platform were fabricated from a commercially available SOI wafer (Soitec) with a 340 nm silicon (Si)/2 µm silicon dioxide (SiO_2)/725 µm Si layer stack, diced into 20×20-mm^2-sized chips. Each chip contained prefabricated 1.5 µm deep markers for later chip alignment in an electron-beam lithography (EBL) tool (EBPG 5200+, Visitec). The standard cleaning procedure for SOI chips consisted of three cleaning steps (each for 3 min): (i) ultrasonication in acetone, (ii) ultrasonication in isopropanol (IPA), and (iii) oxygen plasma cleaning at 600 W (PVA TePla Gigabatch). After cleaning, a 500 nm-thick photoresist mask (AZ1505) was spin coated, baked for 1 min at 110 °C, and exposed under a chrome mask with ultraviolet (UV) light. After exposure, the mask was developed for 20 s in a mixture of AZ400k:H_2O with a ratio of 1:4, rinsed in H_2O, and blow-dried with N_2. The obtained mask contained labels for device numbering and markers for thermal scanning-probe lithography (tSPL) alignment. After developing, the mask was etched with an inductively coupled plasma (ICP) reactive-ion etching (RIE) tool about 60-nm deep into the Si layer[S1] (Oxford Plasmalab System 100). After cleaning, a 260-nm-thick polyphthalaldehyde (PPA, Allresist) layer was spin coated from a 12 wt% solution in anisole (Allresist) and baked for 2 min at 110 °C on a hotplate.

To create the grayscale lithographic mask, the PPA layer on the chip was patterned with tSPL (NanoFrazor® Explore, Heidelberg Instruments), using a new cantilever for each session. As the BR patterns for the optical filters are translationally invariant in the transversal direction, they were chosen to be 8 µm wide. This ensured that they would completely cover the waveguide area without needing a highly precise transversal alignment. The writing time was well under 10 mins including reading of the alignment markers. A dry-etching process [ICP-RIE, C_4F_8/SF_6/argon = 30/20/5 (30/0/5) sccm at 10 (12) mTorr, RF = 15 W, ICP power =1500 (1700) W, where the parentheses indicate the plasma-striking conditions] was used to etch the grayscale patterns from the PPA mask into the Si layer. The typical etch rate in Si was 2.0 nm·s^{-1}, and the etch selectivity was close to PPA:Si = 1:1.1, slightly amplifying the pattern in Si. Note that the etching recipe was sensitive to the DC bias of the ICP-RIE tool. Therefore, before each etching run, test etches were performed and the ICP-RIE parameters were slightly adjusted to ensure a reliable etching process for each chip. We found that a low DC bias between 85 and 90 V was

necessary to keep the etch selectivity of PPA:Si close to 1:1. For a higher DC bias, the etch rate of Si increased quickly.

After a standard cleaning, the sample was then prepared for fabrication of the EBL mask containing the waveguide structures. Here, the chip was cleaned for 5 min in a piranha solution (3:1 H_2SO_4 94%:H_2O_2 30%), rinsed 5 min in deionized water, blow-dried with N_2, and further dried for 10 min on a hotplate at 180 °C. Afterwards, a 180-nm-thick hydrogen silsesquioxane (HSQ 006, Dow Corning) resist layer was spin coated and the integrated photonic circuit was exposed with an EBL tool. The waveguide structures were dry-etched to a depth of 360 nm with ICP-RIE, slightly over-etching the Si layer. Afterwards, the HSQ mask was removed with a buffered hydrofluoric acid solution (BHF, 7:1) for 15 s. For the optical measurements outside of the cleanroom, a 300-nm-thick layer of poly(methyl methacrylate) (PMMA, ARP672.05) was spin coated onto the chip and baked for 5 min at 180 °C to protect the waveguide structures.

Fabrication of lithium-niobate-on-insulator (LNOI) cavities. All devices were fabricated using a commercial LNOI wafer (NanoLN) with an *x*-cut lithium niobate ($LiNbO_3$) device layer. The overall stack consisted of 300 nm $LiNbO_3$/2 µm SiO_2/525 µm Si. The wafer was diced into 15×15 mm^2 chips. Because dry-etching into $LiNbO_3$ is challenging, it was impractical to etch the markers and device labels into the chip. Hence, they were made by depositing metal with a standard double layer lift-off process on the $LiNbO_3$. First, a 300-nm-thick layer of poly(methylmethacrylate-co-methacrylic acid) (PMMA/MA, ARP617.06, Allresist) was spin coated onto the chip and baked for 5 min at 180 °C. Second, a 100-nm-thick layer of PMMA (ARP672.03, Allresist) was spin coated and the chip was baked again for 5 min at 180 °C. After exposure of the obtained bilayer with EBL, it was developed for 2 min in a solution of IPA and methyl isobutyl ketone (MIBK) with a 1:2 ratio and then 1 min in IPA, followed by blow-drying with N_2. A short oxygen plasma clean (200 W for 10 s) was performed to remove potential residues on the $LiNbO_3$ surface. Afterwards, 3 nm of chrome and 120 nm of platinum were evaporated (Evatec BAK501 LL) onto the chip. Finally, the chip was placed for about 5 min into acetone to lift-off the deposited metal on the bilayer, leaving only the EBL markers, tSPL markers, and device labels on the $LiNbO_3$ layer.

A grayscale hard mask for ion milling into $LiNbO_3$ was added. First, a standard cleaning of the chip was performed, and 160 nm of silicon nitride (low stress SiN_x, Oxford PlasmaPro 100

PECVD) was deposited. Afterwards a 280-nm-thick PPA layer (12 wt% solution in anisole) was spin coated and baked for 2 min at 110 °C. At this point, the device labels and tSPL markers were still easily visible through the SiN_x and PPA layers. Their topography could be easily read with the tSPL tool, allowing the alignment of the tSPL patterns. To expose the $LiNbO_3$ layer around the cavities to obtain an undercut later in the process, a deep trench at the transversal border region of the pattern was written (at $y = \pm 5$ µm in Figure S1a). Next, the PPA was etched using an ICP-RIE dry-etching process [C_4F_8/SF_6/argon = 20/5/5 (20/0/5) sccm at 10 (12) mTorr, RF = 16 (20) W, ICP power = 1100 (2000) W, where the parentheses indicate plasma-striking conditions] into the SiN_x layer. The obtained selectivity was PPA:SiN_x = 1:1.4 with a PPA etch rate of 1.1 nm·s^{-1}. Subsequent cleaning of the chip resulted in a SiN_x hard mask containing the grayscale patterns (see Figure S1b). Before the hard mask was etched into $LiNbO_3$, the metallic markers were covered with AZ1512 resist, and the chip was baked for 1 min at 110 °C. Then, the hard mask was milled 145 nm deep into the $LiNbO_3$ layer with an argon-ion milling process (Oxford Ionfab 300, at −20° angle, beam current 500 mA at 600 V, acceleration 390 V, and etch rate of about 18 nm·min^{-1}). This process fully etched the grayscale patterns into $LiNbO_3$, leaving only a thin (sacrificial) SiN_x layer on top of the $LiNbO_3$. Due to charging of the mask and the chip, the argon-milling process was partially isotropic. This reduced the depth of the grayscale pattern in the final $LiNbO_3$ surface (from about 150 nm in the hard mask to about 110 nm in the $LiNbO_3$ layer) and made the peaks in the height profile slightly sharper (Figure S1b). In the future, we are planning to anticipate deviations from the sinusoidal target profile by applying a proximity correction to the tSPL mask. After the ion-milling process, the AZ1512 resist on top of the markers was removed with a 5-min soak in piranha solution. Finally, the remaining SiN_x layer (about 10 nm) was removed with BHF (7:1) for 2 min.

To fabricate the waveguide mask, the chip was again cleaned and a 500 nm thick HSQ (FOX16, Dow Corning) resist layer was spin coated and exposed with EBL. The resulting mask was dry-etched with argon-ion milling to a depth of about 200 nm into the $LiNbO_3$ layer to obtain the ridge waveguides.[S2] Importantly, the trenches at the transversal border region of the pattern were completely etched through the $LiNbO_3$ layer. The lower-lying SiO_2 was exposed by the (i) deep trenches in the original grayscale mask (at $y = \pm 5$ µm, see Figure S1a) and (ii) etched trenches that inherently appear next to the waveguides in the argon-ion milling process (see Figure S1c and ref S2). To remove the redeposited amorphous $LiNbO_3$, the chip was dipped for 20 min into KOH

(44%) at 70 °C. Lastly, the under-etching was performed with a 45-min BHF etch of the chip, which removed the detached $LiNbO_3$ stripes and the residual HSQ mask, as is depicted in Figure S1c.

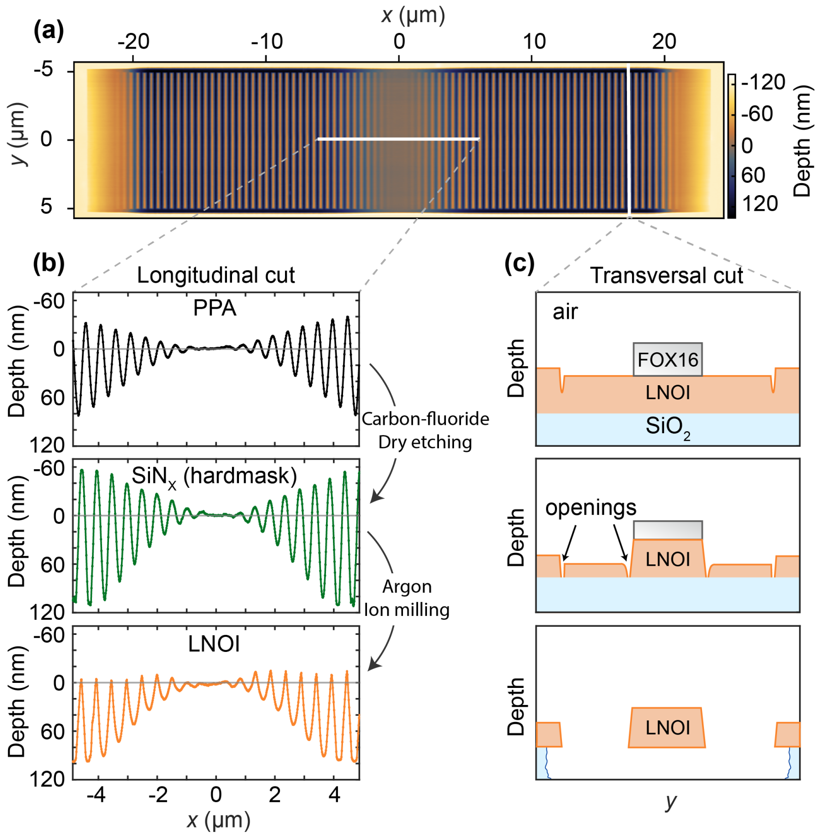

Figure S1. Fabrication scheme of LNOI cavities. (a) Topography of the cavity patterned in PPA recorded with the tSPL tool while writing. Note that the pattern is extended in the transversal (y) direction beyond the waveguide width (1 µm) and contains two deep trenches at $y = \pm 5$ µm. These trenches expose the underlying SiO_2 layer, which enabled the removal of the oxide around the cavity waveguide. (b) Surface profile of the central part of the cavity at different processing steps: PPA mask, SiN_x hard mask after dry etching, and $LiNbO_3$ after argon-ion milling and cleaning. The first profile was taken from the tSPL scan in panel (a), whereas the latter two profiles were recorded with atomic force microscopy. (c) Schematic of the transversal cut during waveguide fabrication with the HSQ mask (FOX16, upper panel). After argon-ion milling, two openings on each side of the waveguide were formed. The outer one came from the trenches in the grayscale pattern; the inner one from the trench effect (middle panel). After the SiO_2 and the HSQ mask were removed, the $LiNbO_3$ waveguide was freestanding in the cavity region (lower panel).

Atomic force microscopy (AFM). The height profiles of the optical Fourier surfaces (OFSs) were characterized with AFM (Dimension Fastscan, Bruker). The sample tilt was corrected in postprocessing via a level plane fit of the flat areas outside the patterned region. Then, a crosscut along the length of the device was taken, which was fit with the desired functional form of the grayscale pattern. The BRs (Figure 2b,e in the main text) consisted of a tangent-hyperbolic envelope superposed on sinusoidal functions. For a BR with a single stop band, the fit function was (following the coordinate system in Figure 1d in the main text)

$$-A\left[\tanh\left(\frac{(z-z_{\text{env}})+w_{\text{env}}}{s_{\text{env}}}\right)+1\right]\left[\sin(k_1 z+\varphi)+1\right]+x\,, \tag{S1}$$

and for a BR with two stop bands, it was

$$-A\left[\tanh\left(\frac{(z-z_{\text{env}})+w_{\text{env}}}{s_{\text{env}}}\right)+1\right]\cdot B\left[(\cos(k_1 z+\varphi)-1)+\left(\cos\left(k_2 z+\varphi+\frac{\pi}{4}\right)-1\right)\right]+x\,, \tag{S2}$$

with A the amplitude of the functional form, z the position, z_{env} the center position of the envelope function, w_{env} the width of the envelope function, s_{env} the slope of the envelope function, B the amplitude of the BR, k_i the spatial frequency of the ith Fourier component, φ the phase, and x the height offset. The topologically protected cavity (Figure 3f) was fit with an exponential envelope function superposed on a sinusoid given by

$$-A\left[1-\exp\left(\frac{z-z_{\text{env}}}{\left(2\sqrt{2}\pi w_{\text{env}}/k_1\right)^2}\right)\right]\cdot\left[1-\cos\left(k_1(z-z_{\text{BR}})+\varphi+\frac{\pi}{2}\left(1-\text{sgn}(z-z_{\text{BR}})\right)\right)\right]+x\,, \tag{S3}$$

with z_{BR} the center position of the BR and sgn the sign function.

Optical characterization. The fabricated devices were characterized by coupling light from a tunable telecom laser with standard telecom fibers into the photonic circuit via one of the fabricated grating couplers. The mode field diameter of the fiber was typically 10.4 µm at 1550 nm. At the end of the circuit, another fabricated grating coupler was used to extract the light from the chip. The transmitted light was then collected with a second telecom fiber and sent to a power meter. For each circuit, the transmitted power as a function of the wavelength was recorded. By comparing each circuit to a reference without the optical device of interest (BR or cavity), the relative transmission was obtained.

When computing the relative transmission in Figure 2c,f in the main text, the noise in the transmission spectra of the reference and device of interest are additive. We assume that this noise, which is due to unwanted reflections, underestimates the actual transmission. Thus, we estimated the actual transmission from a spline fit of the local maxima in the transmission spectra (both of the reference and target devices). The black lines in Figure 2c,f in the main text were then computed from these spline fits. They represent the relative transmission of the device of interest without considering undesired reflections.

Second harmonic generation (SHG) measurements of the LNOI cavities. To measure SHG from the LNOI cavity (see Figure 4 in the main text), the device was pumped with a 1555 nm pulsed laser (Menhir, 18.5 nm bandwidth, 138 fs pulses, 3 kW peak power). Before being coupled into the waveguide through the grating, the light was sent through a variable optical attenuator, then spectrally filtered with a tunable filter, and sent through a 1:99 beam splitter to monitor the average intensity and its wavelength spectrum. A separate optical fiber was brought over the center of the cavity at a vertical distance of about 300 µm. This fiber was attached to an optical spectrometer (Andor Shamrock 303i) to record the emission spectrum.

Numerical simulations. An eigenmode solver (Ansys Lumerical) was used to compute the effective permittivity of the guided modes. Finite-difference time-domain (FDTD) calculations (Ansys Lumerical) were performed to compute the electric fields of the OFS devices in two (2D) and three (3D) dimensions. The modal volume of the cavity was computed from the 3D electric-field distribution of the resonance mode (see eq 1 in ref S3, where we used the refractive index of $LiNbO_3$ along y).

S2. OFS Grating Couplers

Figure S2 shows a scanning electron microscope (SEM) image and atomic force microscope (AFM) scan from a representative grating coupler made in the SOI platform. Because the gratings were not fully optimized in our design, their coupling efficiencies were only ~7.0 % per grating coupler. However, we have previously demonstrated efficiencies of ~45 % for apodized OFS grating couplers in SOI.[S4]

Figure S3 shows SEM and AFM scans of a representative grating coupler made in the LNOI platform. Again, because the gratings were not fully optimized in our design, their coupling

efficiencies were only ~5.4 % per grating coupler. However, 2D FDTD simulations indicate coupling efficiencies of up to 30% within the parameter space accessible to our fabrication methods.

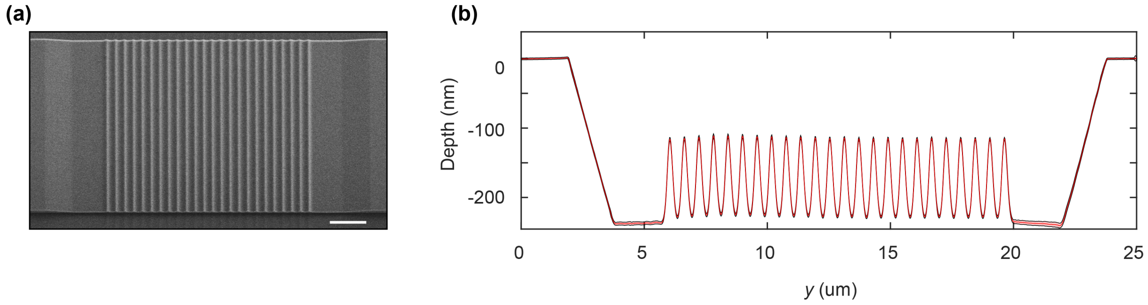

Figure S2. OFS grating coupler in the SOI platform. (a) SEM image of an SOI grating coupler recorded under a 30° angle and 3.0 kV acceleration voltage. The scale bar is 2.5 µm. (b) AFM scan of the same SOI grating coupler. Both the mean (red line) and the standard deviation (shaded area) of multiple scans are shown. The root-mean-square (RMS) error of the sinusoidal region in the center is 6.1 nm (equivalent to 2.5 % of the total depth) with respect to the targeted design.

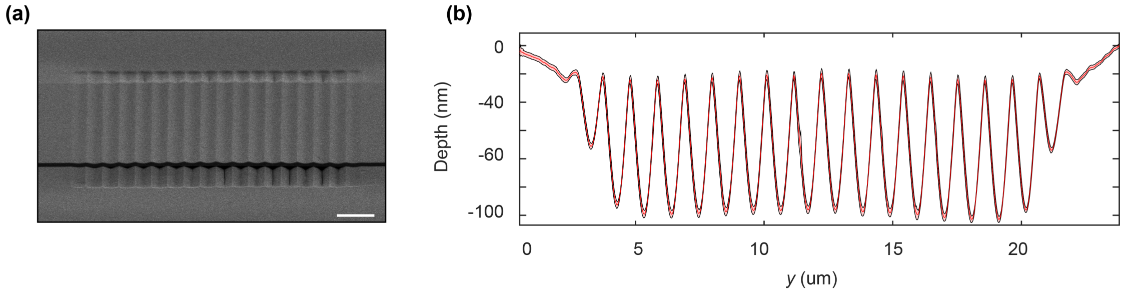

Figure S3. OFS grating coupler in the LNOI platform. (a) SEM image of an LNOI grating coupler recorded under a 70° angle and 3.0 kV acceleration voltage. The scale bar is 2.5 µm. (b) AFM scan of the same LNOI grating coupler. Both the mean (red line) and the standard deviation (shaded area) of multiple scans are shown. The RMS error of the sinusoidal region in the center is 5.8 nm (equivalent to 5.8 % of the total depth) with respect to the targeted design.

S3. Relationship of the Effective Permittivity and Waveguide Height in SOI

To determine when a sinusoidal modulation in height (eq 1 in the main text) leads to a sinusoidal modulation in the relative permittivity (eq 2 in the main text), we performed 2D FDTD calculations. First, we computed the relative effective permittivity, ϵ_{eff}, of the fundamental transverse electric (TE) mode at 1550 nm in a slab Si waveguide cladded with SiO_2. The effective permittivity as a function of the waveguide height h is shown in Figure S4a. The range of the waveguide height in the BRs is shaded in white. Within this range, a sinusoidal modulation in the waveguide height (dashed blue line in Figure S4b) yields an approximate sinusoidal oscillation in the relative effective permittivity (orange line in Figure S4b).

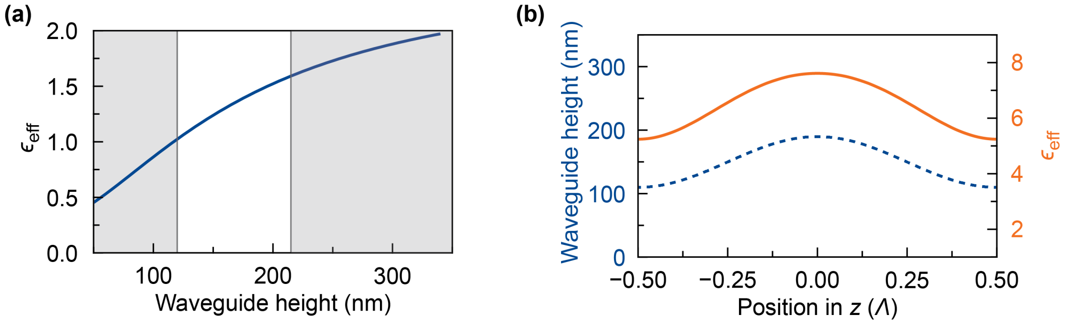

Figure S4. The relationship between permittivity and height in a slab waveguide in SOI. (a) Relative effective permittivity, ϵ_{eff}, of the fundamental mode at 1550 nm in a SOI slab waveguide as function of the Si photonic layer height. The white shaded region corresponds to the depth range of our fabricated BR structures. (b) Unit cell of a sinusoidal height modulation with h_0 = 150 nm and A = 80 nm (dashed blue line, left y-axis) together with the corresponding relative permittivity landscape $\epsilon_{\text{eff},0}$ (orange line, right y-axis).

S4. Model for the Band Structure of the LNOI Cavity

We computed the band structure of a sinusoidally modulated $LiNbO_3$ photonic crystal (Figure 3a in the main text). This model was used to identify starting parameters for the design of the LNOI cavity (final design in Figure 3c in the main text). First, we performed 2D FDTD calculations of the relative effective permittivity, $\epsilon_{\text{eff},0}$, as function of the waveguide height h (see Figure S5a). To describe the BRs, a sinusoidal height profile was defined (eq 1 in the main text with $\varphi = 0$), and the local effective permittivity of the mode was computed via the relationship in Figure S5a. The

obtained relative permittivity landscape ϵ_{eff} is given in Figure S5b and shows, to a good approximation, a sinusoidal form.

Then, we used ϵ_{eff} to calculate the corresponding band structure via the plane-wave-expansion method.[S5] The resulting band diagram is shown in renormalized units in Figure 3a of the main text. For a LNOI cavity with Λ = 544 nm, the calculated bandgap is centered at 1556 nm. This value is close to the resonance wavelength found in the 3D FDTD simulation. The band topology was as-

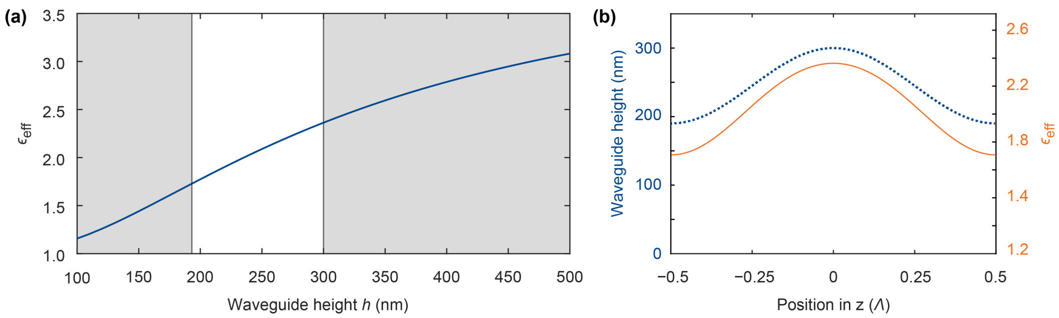

Figure S5. Permittivity and height relationship of $LiNbO_3$ slab waveguides. (a) Relative effective permittivity ϵ_{eff} of the fundamental optical mode at 1550 nm in a 1 µm wide, air-clad LNOI waveguide as a function of the waveguide height h. The white shaded region corresponds to the depth range of our fabricated BR cavity structures. (b) Unit cell of a sinusoidal height modulation with h_0 = 245 nm and A = 55 nm (blue dotted line) together with the corresponding relative permittivity landscape ϵ_{eff} (orange solid line).

signed based on the symmetries of the calculated eigenmodes (Figure 3b in the main text). As the calculations are not restricted to sinusoidal height profiles, any periodic modulation of the waveguide can be modelled. This approach can be used to design photonic band structures for arbitrary periodic OFS devices and to describe its topology.

S5. Optical Properties of the LNOI Cavities

To probe the optical resonance of the LNOI cavities, we use a continuous wave (CW) laser with a narrow linewidth. Due to the high Q of the cavity in Figure 3 of the main text, we observed a typical bistability-type behavior of the resonance at high laser powers (Figure S6). This behavior is the result of thermo-optic heating effects, which tune the cavity resonance along with the CW laser frequency during the measurement.[S6] Therefore, for the characterization of Q, we ensured that low laser powers were used, confirmed by the Lorentzian shape of the resonance.

To further show that the cavity emits frequency-doubled light by second harmonic generation (SHG), we probed the cavity with a broadband fs-laser. The cavity's emitted SHG spectrum is plotted together with the pump light at half its wavelength (Figure S7a). The frequency-doubled light disappeared when the wavelengths around the cavity resonance were removed from the pump spectrum (Figure S7b), showing that the measured SHG was a result of the high Q of the cavity.

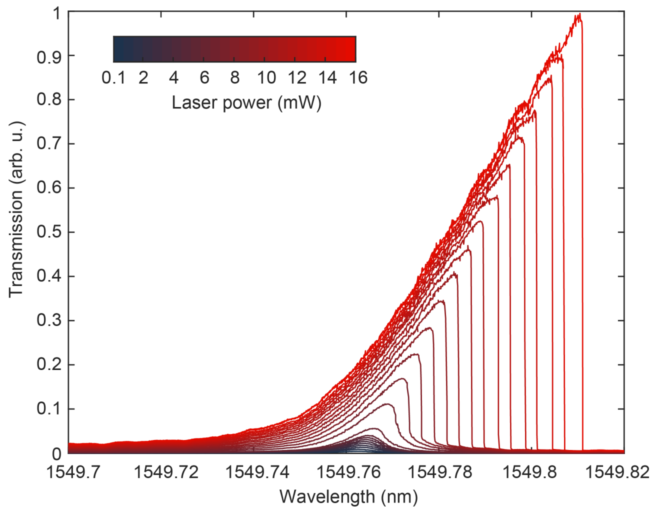

Figure S6. Measured transmission spectra of the high-Q cavity in Figure 3 of the main text, for various powers of the input laser (excluding losses and coupling efficiencies of the overall setup). As the laser power is increased from 0.1 mW to 16 mW, thermal broadening of the cavity resonance is observed, and the resonance shape changes from Lorentzian to a typical bistability-type shape. This is due to thermo-optic heating effects, when the laser was scanned from shorter to longer wavelengths across the resonance at higher powers.

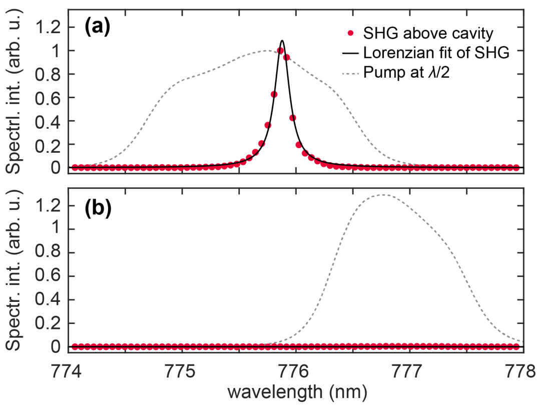

Figure S7. Measured second-harmonic spectrum at different pump wavelengths for the cavity device shown in Figure 4 of the main text. (a) Second harmonic resonance (red dots) plotted with its Lorentzian fit (solid black line). The pump spectrum is resonant with the optical cavity mode at λ_{res} = 1551.64 nm (dashed line, plotted at half the wavelength). (b) When the wavelengths around λ_{res} are removed from the pump light, the SHG disappears.

S6. Supplementary References

(S1) Watanabe, T.; Ayata, M.; Koch, U.; Fedoryshyn, Y.; Leuthold, J. Perpendicular Grating Coupler Based on a Blazed Antiback-Reflection Structure. *J. Light. Technol.* **2017**, *35*, 4663–4669.

(S2) Kaufmann, F.; Finco, G.; Maeder, A.; Grange, R. Redeposition-Free Inductively-Coupled Plasma Etching of Lithium Niobate for Integrated Photonics. *Nanophotonics* **2023**, *12*, 1601–1611.

(S3) Zhou, J.; Zheng, J.; Fang, Z.; Xu, P.; Majumdar, A. Ultra-Low Mode Volume on-Substrate Silicon Nanobeam Cavity. *Opt. Express* **2019**, *27*, 30692–30699.

(S4) Lassaline, N.; Chelladurai, D.; Kohli, M.; Ulrich, R.; Glauser, Y.; Petter, D.; Leuthold, J.; Norris, D. J. Fourier Surface Grating Couplers for Integrated Photonics. *Optica Advanced Photonics Congress 2022* Maastricht, The Netherlands, 24–28 July 2022; p ITh2B.5.

(S5) Joannopoulos, J. D.; Johnson, S. G.; Winn, J. N.; Meade, R. D. *Photonic Crystals: Molding the Flow of Light*, 2nd ed.; Princeton University Press, 2008.

(S6) Liang, H.; Luo, R.; He, Y.; Jiang, H.; Lin, Q. High-Quality Lithium Niobate Photonic Crystal Nanocavities. *Optica* **2017**, *4*, 1251–1258.
